# Smoking prevalence in Italy from 1993 to 2024: a trend analysis framed by public health policies and emerging consumption behaviors

**DOI:** 10.1093/pubmed/fdag055

**Published:** 2026-07-01

**Authors:** Michele Bonacquisti, Luigi Russo, Maria Rosaria Gualano, Walter Ricciardi, Leonardo Villani

**Affiliations:** Section of Hygiene–University Department of Life Sciences and Public Health, Università Cattolica Del Sacro Cuore, Largo Francesco Vito, 1, 00168 Rome, RM, Italy; Section of Hygiene–University Department of Life Sciences and Public Health, Università Cattolica Del Sacro Cuore, Largo Francesco Vito, 1, 00168 Rome, RM, Italy; UniCamillus–Saint Camillus International University of Health and Medical Sciences, Largo Francesco Vito, 1, 00168 Rome, RM, Italy; Section of Hygiene–University Department of Life Sciences and Public Health, Università Cattolica Del Sacro Cuore, Largo Francesco Vito, 1, 00168 Rome, RM, Italy; Section of Hygiene–University Department of Life Sciences and Public Health, Università Cattolica Del Sacro Cuore, Largo Francesco Vito, 1, 00168 Rome, RM, Italy; UniCamillus–Saint Camillus International University of Health and Medical Sciences, Largo Francesco Vito, 1, 00168 Rome, RM, Italy

**Keywords:** smoking, electronic nicotine delivery devices, prevention, public policy, surveillance and monitoring, secondhand smoke

## Abstract

**Background:**

Smoking remains a major public health issue in Italy, despite decades of tobacco control measures. This study aimed to describe trends in smoking prevalence and in consumption of other tobacco-derived products, in Italy from 1993 to 2024 stratified by gender and age group.

**Methods:**

This is an observational ecological time-series analysis. Smoking prevalence data were obtained from the Italian Institute of Statistics. Joinpoint regression model was applied to estimate the Annual Percentage Change (APC) and the Average Annual Percentage Change (AAPC). Data on tobacco-derived products were analyzed descriptively using data from the Italian National Health Institute (2013–2023).

**Results:**

Overall smoking prevalence declined significantly over the study period (AAPC = −0.78%; *P* < .0001), with a greater reduction among men (AAPC = −1.31%; *P* < .0001). However, an increase is observed from 2020 to 2024 (APC +1.53; *P* = .009). Long-term reductions were observed among midlife women, while smoking increased among younger and older women. E-cigarette use declined (−40%), while heated tobacco products and hand rolled cigarettes use rose steadily (+236% and +181%, respectively).

**Conclusions:**

The recent rise in smoking and the growing use of alternative nicotine products demand renewed prevention and cessation efforts, with targeted education and stronger regulation.

## Introduction

Tobacco consumption represents one of the greatest challenges to public health worldwide, contributing to more than 8 million deaths each year, including approximately 1.3 million attributable to exposure to secondhand smoke.^1^ Within the European Union, despite variations across Member States, smoking is responsible for around 700 000 deaths annually.^2^ In Italy, tobacco use accounts for more than 96 000 deaths per year, with a high prevalence of smokers and significant costs estimated at over €23 billion.^3^

Several historical, cultural, and socioeconomic factors have contributed to Italy’s persistently high smoking prevalence. Throughout the 20th century, tobacco use was deeply embedded in Italian social and cultural life, and associated with leisure, sociability, and modernity.^4^

Widespread smoking was also favored by the strong presence of the tobacco industry, the relatively low cost of cigarettes,^5^ limited awareness of health risks, and weak anti-smoking policies, especially before the early 2000s. Smoking was normalized in public spaces, such as cafés and restaurants, further contributing to its social acceptance and intergenerational transmission.^6^

Over the years, the Italian government has implemented several measures to reduce smoking prevalence, including awareness campaigns, fiscal and legislative measures.^6–9^ Among these, the Law 3/2003 (Art. 51), titled ‘Protection of Non-Smokers’ Health’, came into force in 2005, and banned smoking in all enclosed public spaces and workplaces, aiming to reduce secondhand smoke exposure.^10^ Similarly, in 2003 Italy signed the World Health Organization Framework Convention on Tobacco Control (WHO FCTC),^11^ ratified in 2008, that establishes goals and principles for the reduction of tobacco use.^12^

Despite these advances, tobacco consumption remains a persistent problem in Italy. Socioeconomic factors also influence smoking behavior: individuals with lower levels of education and income show higher smoking rates^13^ and are more at risk of both active and passive smoking.^14^^,^^15^ Although national surveillance systems routinely provide data on smoking prevalence, analyses that examine trends by gender and age group, while also considering the evolving landscape of tobacco-derived products within the context of major policy developments, remain relatively limited. In this context, monitoring temporal variations is essential to assess differences by gender and age and to inform targeted public health interventions.

The aim of this study is to describe trends in smoking prevalence in Italy, stratified by gender and age group, and to analyse changes in the consumption of cigarettes and other tobacco-derived products in the context of national policies and evolving consumer behaviors.

## Methods

This study is an observational ecological time-series analysis based on nationally aggregated data and is reported in accordance with the STROBE guidelines.^16^

### Definitions

Smoking prevalence refers to the proportion of individuals who smoke manufactured tobacco cigarettes. The smoking status in the National survey was assessed through self-report and defined as the percentage of individuals within a given age and sex group who reported smoking habitually, calculated as the ratio between habitual smokers and the corresponding population subgroup ×100.

Other forms of tobacco or nicotine use are reported separately and include electronic cigarettes (e-cigarettes), hand-rolled cigarettes (roll-your-own, RYO), and heated tobacco products (HTPs). In this case, the smoking status (current smoker, occasional smoker, daily smoker, ex-smoker, and recent quitter) was defined according to the operational criteria used in the Italian National Health Institute surveillance systems. The reported values represent nationally representative prevalence estimates derived from the survey methodology and are presented as crude percentages unless otherwise specified.

### Data sources and collection on the prevalence of smokers

The data on the proportion of smokers in Italy were obtained from the Italian Institute of Statistics (ISTAT), which surveys a representative sample of approximately 24 000 individuals each year as part of the questionnaire *‘Indagine multiscopo sulle famiglie – Aspetti della vita quotidiana’,*^17^ and public available at Istat Health For All.^18^ The survey is based on a probabilistic sampling design stratified by geographic area and municipality size, ensuring representativeness at both national and regional levels. The questionnaire is administered to household members using standardized survey procedures, and published estimates are weighted to represent the Italian population. Thus, the analyses relied on weighted, nationally representative aggregated estimates. No major methodological breaks affecting this indicator are documented in the official sources, while missing data were handled by ISTAT according to standard data quality procedures. Similarly, the definition of habitual smoker remained consistent over the study period.^19^ The dataset covers the years from 1993 to 2024, excluding 2004 due to unavailability, stratified by gender and age, with the following age groups: 15–24, 25–34, 35–44, 45–54, 55–64, and ≥ 65.

### Data sources and collection on the consumption of other forms of tobacco or nicotine

Data on consumption in Italy of e-cigarettes, RYO, and HTPs were obtained from the Italian National Health Institute.^20^

Data were derived from the national cross-sectional survey conducted jointly by the Italian National Health Institute and the Istituto Mario Negri. The survey is based on a sample representative of the Italian population aged ≥15 years, selected according to the main sociodemographic characteristics. Data are collected through anonymous questionnaires administered using Computer Assisted Personal Interview methodology.

The dataset for these covers different timespans: e-cigarettes (2013–2023), HTPs (2019–2023), and RYO (2010–2023).

Smoking status was assessed through self-report and defined as the percentage of individuals within a given age and sex group who reported smoking habitually (‘persone che fumano abitualmente’), calculated as the ratio between habitual smokers and the corresponding population subgroup ×100. According to ISTAT metadata, the definition of habitual smoker remained consistent over the study period (1993–2003; 2005–2024), although minor changes in questionnaire wording or survey procedures cannot be entirely excluded.

### Statistical analysis

We conducted descriptive analyses of smoking trends, stratified by gender and age group, evaluating smoking prevalence per year.

Furthermore, to evaluate trends related to the prevalence of smokers, we conducted a Joinpoint regression analysis. A joinpoint model examines time series by fitting segmented linear trends, identifying breakpoints (joinpoints), in which a change in the trend occurs.^21^ We used the Joinpoint Regression Program (NCI) with default settings. The maximum number of joinpoints (5) was determined automatically by the software based on the length of the time series. The final model was selected using the permutation test procedure implemented in the program (overall two-sided α = 0.05), choosing the most parsimonious model that provided a statistically significant improvement in fit. A logarithmic transformation was applied to the annual percentages of smokers to estimate the Annual Percentage Change (APC) assuming constant relative changes over time. The rate of change in each segment is quantified by the APC, which indicates how quickly the outcome increases or decreases on a yearly basis in that specific interval. The Average Annual Percentage Change (AAPC) summarizes the overall trend across the entire period by taking an average of the APCs.

Residual of the time series were found to be homoscedastic and normally distributed. First-order autocorrelation was addressed using the Joinpoint software.

For the analysis on the use of e-cigarettes, HTPs, and RYO, since data was available only for a short period of time, it was not possible to perform a joinpoint analysis. To allow comparisons and evaluate differences across the examined period, descriptive analyses were conducted to examine the percentage variations, from the first available year to the most recent, for each category.

All the data were analyzed using Stata 18^22^ and Joinpoint Software 5.4.^23^

## Results

### Descriptive analysis of smoking prevalence in Italy by gender and age

From 1993 to 2024, the prevalence of smokers in Italy ranged, in the overall population, from 18.63% in 2019 to 26.37% in 1996, in males from 22.54% in 2020 to 33.58% in 1997, and in females from 14.85% in 2019 to 18.03% in 1996.

Similarities in the two gender groups emerged, as the highest prevalence was observed for the 35–44-year age group for both sexes, 45.13% in 1995 for males and 29.69% in 1996 for females, while the lowest was observed in the ≥65-year group, with 11.24% in 2017 for males and 4.79% in 2003 for females. Notably, the highest value of the ≥65-year group of 22.85%, observed in 1993, is lower than the lowest ever registered in the 35–44 age group (28.07% in 2020).

Regarding the last available year, a similar age gradient in both sexes was observed, with a prevalence of 31.87% for men and 22.31% for women, both in the 35–44-year age group and 13.33% in men and 9.09% in women, in the ≥65-year group. Data on the prevalence of smokers in Italy are reported in Table 1.

**Table 1:** Prevalence of smokers (overall data and stratified by gender and age) in Italy from 1993 to 2024.

Gender	Male	Female	Male	Female	Male	Female	Male	Female	Male	Female	Male	Female	Male	Female	Male and Female
Age Year	15–24		25–34		35–44		45–54		55–64		65+		Total		
**1993**	27.16	13.41	41.93^*^	24.55	**43.36** ^**#**^	**25.94** ^**#**^	42.21^*^	21.19	35.20^*^	12.42	22.85^*^	4.92	35.58^*^	16.62	25.75
**1994**	26.42	12.90	41.24	25.10	**44.48** ^**#**^	**26.73** ^**#**^	40.60	20.55	31.89	12.65	21.17	5.80	34.49	16.88	25.36
**1995**	27.24	13.21	40.46	25.41^*^	***45.13*** ^#^*^^	**27.79** ^**#**^	41.08	21.71	30.51	13.48	20.36	5.90	34.38	17.43	25.58
**1996**	30.44^*^	15.05	41.69	23.92	**42.88** ^**#**^	***29.69*** ^#^*^^	42.05	23.87	34.29	13.27	19.87	5.77	35.35	18.03^*^	26.37^*^
**1997**	29.38	14.78	**40.74** ^**#**^	23.02	40.60	**28.18** ^**#**^	38.39	21.74	31.54	14.23	19.48	5.97	33.58	17.46	25.22
**1998**	28.92	16.93	38.67	23.14	**40.49** ^**#**^	**27.78** ^**#**^	37.46	22.39	30.20	13.10	18.21	5.29	32.59	17.46	24.73
**1999**	29.66	16.54	**39.84** ^**#**^	20.84	38.68	**27.99** ^**#**^	39.10	21.18	30.93	15.07	17.30	5.95	32.78	17.28	24.74
**2000**	27.15	16.42	**39.14** ^**#**^	21.17	37.31	**27.68** ^**#**^	38.38	24.00	29.95	13.81	17.78	5.25	31.86	17.42	24.38
**2001**	27.13	15.93	**38.66** ^**#**^	20.85	38.31	**25.68** ^**#**^	37.13	22.44	29.14	16.06	17.32	5.67	31.57	17.09	24.07
**2002**	27.22	16.78	**39.37** ^**#**^	21.62	38.42	**24.86** ^**#**^	36.43	24.19	28.39	13.80	16.25	6.26	31.26	17.24	23.99
**2003**	26.43	17.77	**39.28** ^**#**^	22.02	37.32	**25.00** ^**#**^	36.50	24.34	29.76	16.89	17.57	4.79	31.36	17.56	24.22
**2005**	27.11	16.40	**36.51** ^**#**^	21.09	34.12	22.18	32.99	**22.53** ^**#**^	27.65	15.08	14.31	6.11	28.66	16.38	22.29
**2006**	25.68	16.86	**36.93** ^**#**^	21.94	36.60	22.66	33.36	**24.35** ^**#**^	28.40	18.41	14.49	5.68	29.17	17.22	22.98
**2007**	25.53	15.74	**38.00** ^**#**^	22.09	35.03	21.30	34.00	**23.11** ^**#**^	24.80	17.37	14.58	6.16	28.59	16.63	22.39
**2008**	27.29	15.75	**36.89** ^**#**^	21.0**4**	35.45	20.03	32.97	**24.30** ^**#**^	29.07	17.54	13.55	6.09	28.89	16.43	22.43
**2009**	26.11	16.95	**40.17** ^**#**^	22.24	35.10	20.77	34.04	***24.54*** ^#^*^^	29.81	18.31	15.58	6.47	29.87	17.13	23.27
**2010**	27.37	15.45	**39.72** ^**#**^	24.42	36.73	18.97	34.03	**24.45** ^**#**^	27.45	18.25	14.15	7.16	29.55	17.06	23.07
**2011**	26.48	15.91	**38.86** ^**#**^	22.44	35.24	19.63	32.13	**23.26** ^**#**^	27.90	18.83	14.30	6.66	28.72	16.75	22.52
**2012**	25.48	15.57	**35.88** ^**#**^	21.30	35.05	19.5	33.92	**23.44** ^**#**^	26.71	18.43	14.11	6.84	28.13	16.49	22.10
**2013**	25.50	14.75	**36.22** ^**#**^	20.4	32.68	19.31	31.68	**22.06** ^**#**^	25.25	18.11	12.56	6.65	26.66	15.90	21.08
**2014**	22.18	14.43	**33.50** ^**#**^	19.25	31.50	18.60	27.97	**20.32** ^**#**^	25.12	16.66	12.21	6.17	24.78	14.91	19.66
**2015**	22.40	14.03	**32.97** ^**#**^	19.37	31.27	17.46	29.47	**19.70** ^**#**^	25.16	18.73	11.97	7.30	24.85	15.15	19.82
**2016**	20.98	15.68	**33.45** ^**#**^	19.06	32.19	18.41	29.80	**19.07** ^**#**^	24.23	19.09	13.44	6.98	25.08	15.27	19.99
**2017**	20.86	14.65	33.15	17.82	**35.64** ^**#**^	18.51	30.66	**20.40** ^**#**^	23.44	17.74	11.24	6.94	25.08	15.02	19.87
**2018**	23.52	15.68	**32.04** ^**#**^	17.89	30.29	17.79	26.27	**19.06** ^**#**^	22.79	18.70	12.04	7.74	23.51	15.13	19.18
**2019**	20.40	13.32	**29.99** ^**#**^	17.16	29.24	**19.08** ^**#**^	25.89	17.96	23.20	17.35	12.36	8.78	22.68	14.85	18.63
**2020**	20.78	13.49	**29.86** ^**#**^	18.26	28.07	18.18	26.54	**18.86** ^**#**^	23.98	19.81^*^	11.84	8.77	22.54	15.37	18.83
**2021**	19.00	14.99	**31.49** ^**#**^	**19.65** ^**#**^	30.40	19.26	27.88	18.31	22.64	18.63	12.66	8.60	23.06	15.48	19.14
**2022**	20.58	12.86	32.60	19.44	**33.72** ^**#**^	**20.66** ^**#**^	27.91	19.05	23.20	20.12	13.41	8.56	24.07	15.75	19.77
**2023**	20.46	16.18	**33.92** ^**#**^	**19.58** ^**#**^	30.87	19.22	28.35	18.90	21.59	19.62	12.68	8.76	23.35	15.84	19.48
**2024**	21.06	17.82^*^	31.42	19.66	**31.87** ^**#**^	**22.31** ^**#**^	27.64	20.34	23.07	18.93	13.33	9.09^*^	23.50	16.64	19.97

In bold and # the highest value per year for men and women; in italics and ^*^ the year with the highest value for each age group in men and women. Data for 2004 were not available.

**Figure 1:**
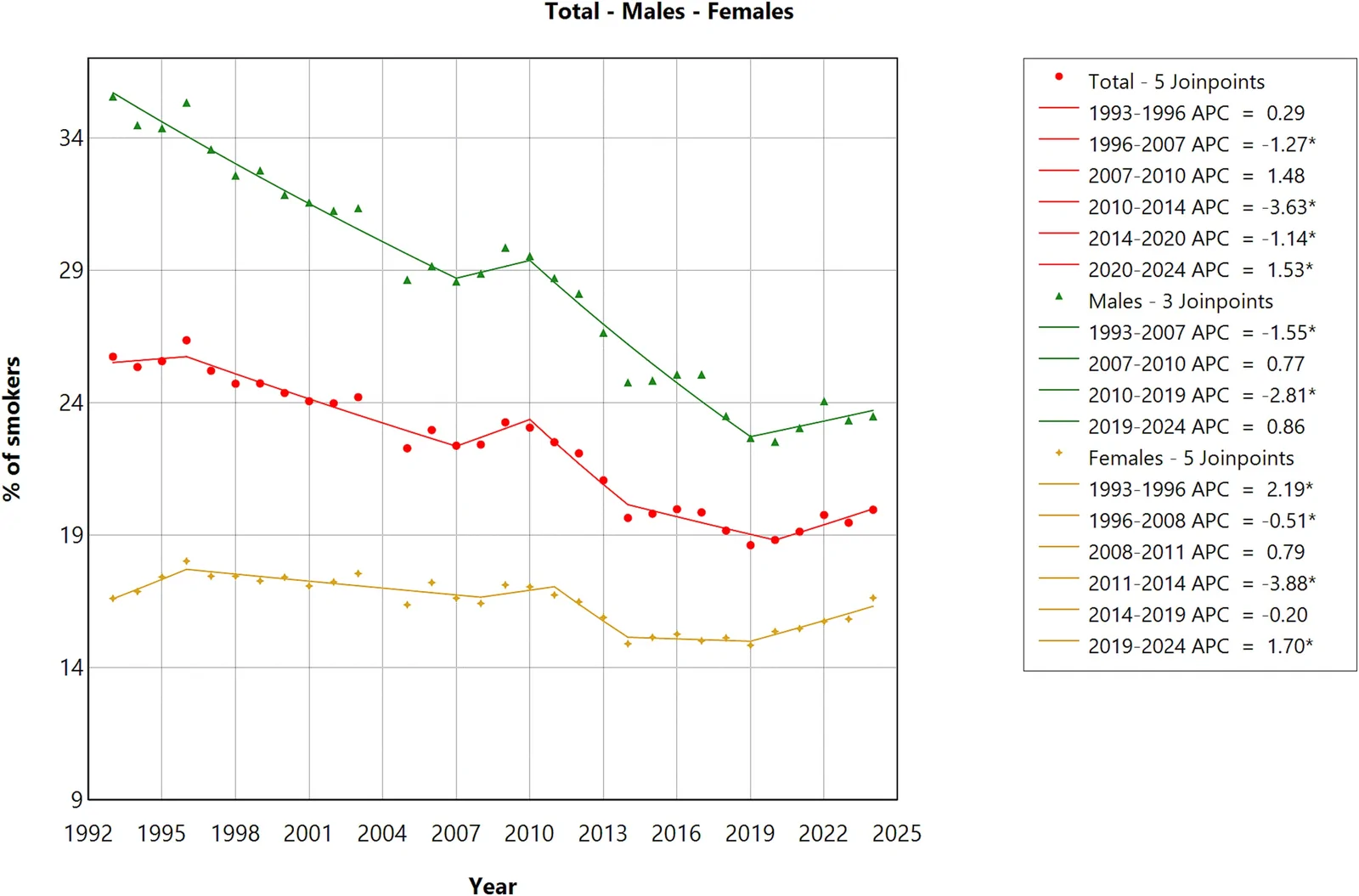
Trends of percentage of smokers (overall data and data stratified by gender) in Italy from 1993 to 2024.

### Relevant changes in trends by gender and age groups, from 1993 to 2024

Overall, a reduction was observed for both sexes and across all stratified age groups, while several changes occurred during the study period.

In the overall population, multiple significant segments of decrease were identified (between 1996 and 2007, 2010 and 2014, and 2014 and 2020) followed by a significant increase between 2020 and 2024 (APC = +1.53; 95% CI: 0.46 to 3.72; *P* = .008). A similar pattern was found in the overall gender-stratified analysis, with multiple decreasing trends followed by a significant increase among females between 2019 and 2024 (APC = +1.70; 95% CI: 1.10 to 3.02; *P* = .004), while the rise was not significant among males (Fig. 1, Table 2).

**Figure 2:**
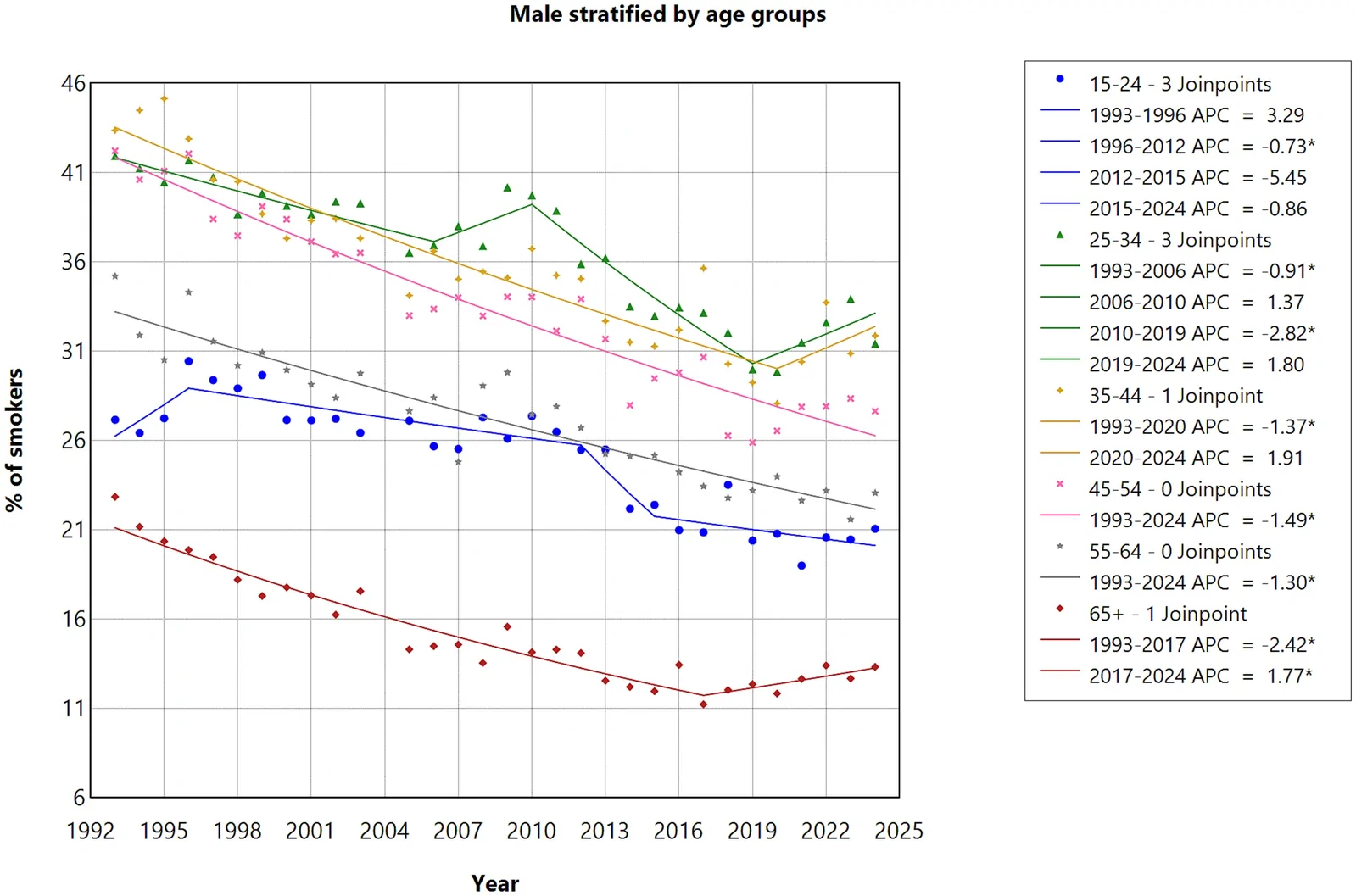
Trends of prevalence of male smokers (stratified by age groups) in Italy from 1993 to 2024.

Regarding the most recent trends, the male population shows a non-significant increase in the APC across all age groups, except for the 15–24 group. A significant increase was observed in the ≥65 group from 2017 to 2024 (APC = +1.77%; 95% CI: 0.27 to 4.69; *P* = .01) (Fig. 2).

**Figure 3:**
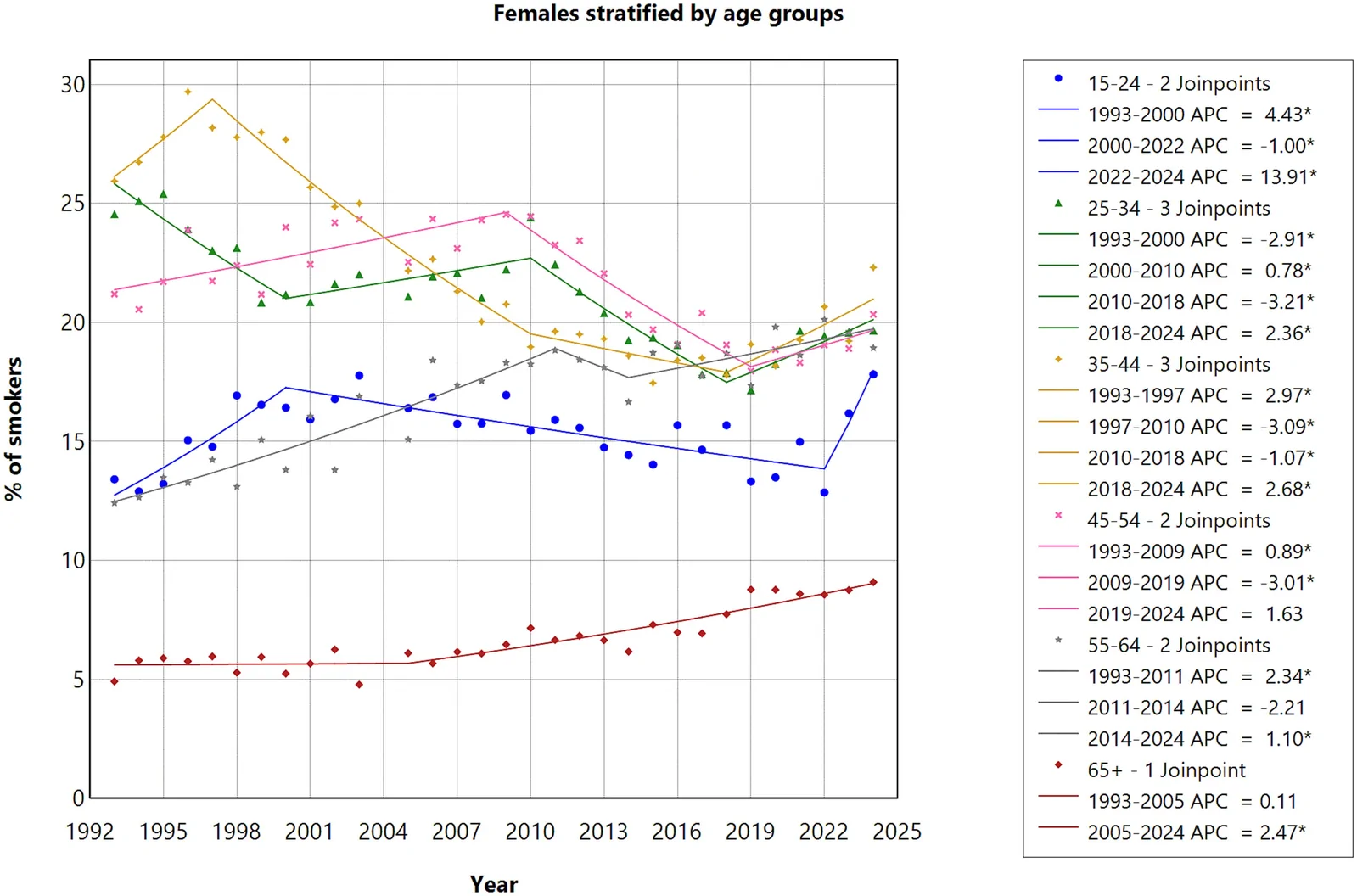
Time trends of prevalence of female smokers (stratified by age groups) in Italy from 1993 to 2024.

The most recent trends for the female population show a significant increase in smoking prevalence in almost all age groups between 2022 and 2024 (Fig. 3). The APC increased in the 25–34 and 35–44 age groups between 2018 and 2024, in the 55–64 age group between 2014 and 2024, and in the ≥65 age group between 2005 and 2024. A non-significant increase was also observed in the 45–54 age group. Details on the APC changes are shown in Table 2.

### Trends analysis by gender and age groups, from 1993 to 2024

Overall, the joinpoint analysis highlighted a significant reduction between 1993 and 2024 (Table 3) in smoking prevalence in the whole population (AAPC = −0.78%; *P* < .0001).

Among the male population, a significant reduction was observed (AAPC = −1.31%; *P* < .0001), and similar reductions were observed in the age-stratified analysis: 15–24 years (AAPC = −0.85; *P* < .0001), 25–34 years (AAPC = −0.75; *P* < .0001), 35–44 years (AAPC = −0.95; *P* < .0001), 45–54 years (AAPC = −1.49; *P* < .0001), 55–64 years (AAPC = −1.30; *P* < .0001), and ≥ 65 years (AAPC = −1.49; *P* < .0001) (Fig. 2, Table 3).

Among the female population, a slight, non-significant decrease was reported (AAPC = −0.055%; *P* = .18). Distinct patterns were observed across age groups. A significant increase was recorded specifically among women aged 15–24 (AAPC = +1.11; *P* < .000001), 55–64 (AAPC = +1.49; *P* < .000001), and ≥ 65 years (AAPC = +1.55; *P* < .000001). Significant reductions, instead, were observed in the 25–34 (AAPC = −0.80; *P* < .000001), 35–44 (AAPC = −0.70; *P* < .000001), and 45–54 age groups (AAPC = −0.27; *P* = .01) (Fig. 3, Table 3).

### Descriptive analysis of alternative tobacco and nicotine product use

Patterns of alternative tobacco product use evolved over time (Table 4). The proportion of exclusive e-cigarette users declined from 4.2% in 2013 to 2.5% in 2023 (Δ = −40%). After an initial decrease, dual use (tobacco + e-cigarettes) rebounded in 2023 to values comparable to those observed in 2013 (85.6% vs. 89.4%; Δ = −4.3%). The prevalence of both HTP and RYO use has risen in recent years. HTP use increased from 1.1% in 2019 to 3.7% in 2023 (Δ = +236%), while RYO use grew from 2.6% in 2010 to 7.3% in 2023 (+181%), peaking at 10.8% in 2022.

**Table 2:** APC stratified by gender and cohort from 1993 to 2024.

Cohort	Gender	First year	Last year	APC	Lower CI	Upper CI	*P*-Value
15–24	Females	1993	2000	4.4261^*^	29.641	66.501	**.0012**
		2000	2022	−0.9961^*^	−1406	−0.7257	**.0012**
		2022	2024	13.9087^*^	35.773	18.7517	**.004399**
	Males	1993	1996	32.859	−0.4651	10.4383	.156369
		1996	2012	−0.7256^*^	−64.527	−0.3232	**.023195**
		2012	2015	−54.539	−71.412	32.051	.191562
		2015	2024	−0.8615	−49.466	29.842	.343931
25–34	Females	1993	2000	−2.9050^*^	−44.399	−19.463	**< .000001**
		2000	2010	0.7764^*^	0.1751	22.416	**.010798**
		2010	2018	−3.2078^*^	−49.203	−23.779	**< .000001**
		2018	2024	2.3632^*^	10.973	42.227	**< .000001**
	Males	1993	2006	−0.9125^*^	−26.148	−0.4268	**.031594**
		2006	2010	13.693	−19.486	33.483	.260748
		2010	2019	−2.8230^*^	−54.806	−19.271	**.020796**
		2019	2024	17.951	−0.0583	56.952	.057189
35–44	Females	1993	1997	2.9685^*^	12.918	56.212	**.0012**
		1997	2010	−3.0944^*^	−37.165	−28.076	**< .000001**
		2010	2018	−1.0706^*^	−19.726	−0.0767	**.039592**
		2018	2024	2.6764^*^	16.851	4535	**< .000001**
	Males	1993	2020	−1.3655^*^	−23.399	−11.384	**.019596**
		2020	2024	19.075	−11.111	85.777	.293141
45–54	Females	1993	2009	0.8888^*^	0.5558	12.761	**.0008**
		2009	2019	−3.0073^*^	−44.882	−23.457	**.0012**
		2019	2024	16.295	−0.2587	56.596	.095181
	Males	1993	2024	−1.4903^*^	−16.675	−13.087	**< .000001**
55–64	Females	1993	2011	2.3382^*^	21.399	25.535	**.003599**
		2011	2014	−22.056	−31.565	0.2546	.087982
		2014	2024	1.0985^*^	0.6585	23.415	**.0016**
	Males	1993	2024	−1.2964^*^	−14.337	−11.559	**< .000001**
65+	Females	1993	2005	0.1073	−15.653	0.9866	.862228
		2005	2024	2.4723^*^	20.249	32.066	**< .000001**
	Males	1993	2017	−2.4168^*^	−27.016	−21.794	**< .000001**
		2017	2024	1.7712^*^	0.2706	46.996	**.019196**
Total	Females	1993	1996	2.1854^*^	10.206	41.085	**.000000**
		1996	2008	−0.5087^*^	−11.442	−0.3723	**.004799**
		2008	2011	0.787	−0.3051	13.413	.15037
		2011	2014	−3.8814^*^	−45.119	−24.013	**.023595**
		2014	2019	−0.1976	−11.051	0.6605	.579484
		2019	2024	1.7021^*^	11.037	30.245	**.004399**
	Males	1993	2007	−1.5469^*^	−26.391	−1269	**.016797**
		2007	2010	0.7749	−13.539	18.363	.658668
		2010	2019	−2.8140^*^	−45.171	−21.931	**.012797**
		2019	2024	0.8589	−0.4276	3.53	.173565
	Males and females	1993	1996	0.2876	−0.8975	25.267	.75065
		1996	2007	−1.2722^*^	−24.625	−10.751	**.027594**
		2007	2010	14.837	−0.5773	2403	.119176
		2010	2014	−3.6347^*^	−48.413	−2295	**.023195**
		2014	2020	−1.1377^*^	−21.772	−0.1256	**.035193**
		2020	2024	1.5296^*^	0.4616	37.286	**.007598**

**Table 3:** AAPC stratified by gender and age group from 1993 to 2024.

Gender	Age group	AAPC	Lower CI	Upper CI	*P*-Value
Male	15–24	−0.9078	−1.26	−0.5541	**< .000001**
Female		+0.3739	0.1321	0.6978	.003999
Male	25–34	−0.6871	−0.8134	−0.5671	**< .000001**
Female		−0.8635	−1.0678	−0.6627	**< .000001**
Male	35–44	−0.9810	−1.4357	−0.6736	**< .000001**
Female		−0.9096	−1.0975	−0.6898	**< .000001**
Male	45–54	−1.5247	−1.6993	−1.3354	**< .000001**
Female		−0.4991	−0.6839	−0.2932	**< .000001**
Male	55–64	−1.3103	−1.4688	−1.1448	**< .000001**
Female		+1.5346	1.4316	1.6714	**< .000001**
Male	≥ 65	−1.5833	−1.8683	−1.3128	**< .000001**
Female		+1.5183	1.2029	1.8389	**< .000001**
Male	Overall	−1.3114	−14.602	−11.794	**< .000001**
Female		−0.0551	−0.1319	0.0386	.175965
Total		−0.7842	−0.8928	−0.6488	**< .000001**

**Table 4:** Percentage of different types of smokers in Italy from 2010 to 2023.

Year	e-cig smokers (esclusive)	e-cig smokers (dual user)	HTPs smokers	RYO smokers
2010	NA	NA	NA	2.6
2011	NA	NA	NA	3.4
2012	NA	NA	NA	5.9
2013	4.2	89.4	NA	6
2014	1.6	80.7	NA	7.8
2015	1.1	73.1	NA	6.9
2016	3.9	77.6	NA	9.4
2017	2.5	83.4	NA	9.6
2018	2.1	75.3	NA	9.4
2019	1.7	79.5	1.1	10.4
2022	2.4	81.9	3.3	10.8
2023	2.5	85.6	3.7	7.3
Δ 2010–2023	−40.5%	−4.3%	+236.4%	+180.8%

NA, data not available; e-cig, electronic cigarettes; HTPs, heated tobacco products; RYO, hand-rolled cigarettes.

## Discussion

### Main findings of the study

Our work highlights an overall decline in smoking prevalence over the past 30 years in Italy, though with marked differences by period, gender, and age group. Distinct patterns emerged: smoking increased among women aged 15–24, 55–64, and ≥ 65 throughout the study period, consistent with a rise in all female age groups in recent years, as well as in young men (25–44 years) and men aged ≥65.

After a marked decline since the mid-1990s, smoking prevalence recently increased again, possibly reflecting evolving consumption habits and the spread of alternative nicotine products. Our analysis highlighted differences among age groups, with the youngest and oldest groups tended to behave similarly: in recent years, prevalence rose notably among young adults and adults aged ≥65.

### What is already known on this topic

Gender differences in smoking are well established, both motivationally and biologically.^24^^,^^25^ Women historically show about a generation lag compared to men, explaining opposite trends across equivalent age groups.^26^ Education has also shaped female smoking patterns: while in the 1990s mostly highly educated women smoked, this relationship reversed after 2000, aligning more closely with men.^13^ The COVID-19 pandemic may also have had a role in modifying smoking behaviors. Psychological distress is well known to facilitate smoking initiation or relapse,^27^^,^^28^ which is consistent with observed general increases in prevalence during the pandemic.^29–38^

Smoking initiation typically occurs at a young age,^30^^,^^39^ and may be linked to limited awareness of health risks,^31^ new nicotine products,^32^ and dual use.^33^^,^^34^ Particularly, the aggressiveness of nicotine substitutes, including marketing strategies, affordability, and the visually appealing design features (e.g., colors, flavors) represent a new public health challenge.^35–37^

Smoking determinants in older adults remain multifactorial,^38^ including health, social and economic status, psychological factors, and misconceptions about cessation, all of which impede cessation efforts. Nonetheless, this population is often considered receptive to quitting,^40^ especially after new diagnoses of chronic illness^41^ or medical advice.^42^

### What this study adds

Our study is the first to analyse trends in the prevalence of smokers and users of other tobacco and nicotine products in Italy over a period of more than 30 years, by gender and age group.

We found that smoking rates among adults aged ≥65 declined steadily until a slight increase is observed in recent years among men, while, on the contrary, female prevalence has risen continuously since 2005, indicating a progressive normalization of smoking within this cohort.^43^

Moreover, emerging use patterns show heterogeneous trends: e-cigarette use has declined, while HTPs continue to grow. Both are widely perceived as less harmful than traditional cigarettes, an idea reinforced by social exposure and advertising.^44^^,^^45^ Moreover, given the persistence of dual use in Italy,^46^^,^^47^ their effectiveness as cessation tools is limited.^48^^,^^49^

Public policies have played a key role in controlling tobacco use. The WHO FCTC, adopted in 2003^11^ and ratified in Italy in 2008,^50^ laid the foundation for a regulatory framework. The 2003 National Law^6^ significantly reduced smoking and secondhand exposure, and although Joinpoint regression does not allow direct causal inference, a clear downward trend in prevalence was observed. Legislation has evolved over time, first extending to e-cigarettes in 2013,^51^ and later to HTPs in 2016.^52^ In 2019, tobacco product sponsorship was banned on social media platforms.^53^

Finally, there is scope for improvement in Italy concerning the involvement of the tobacco industry in policy development, transparency, management of conflicts of interest, and the reduction of unnecessary interactions.^54^ Italy, indeed, lacks any legal framework restricting tobacco industry involvement in public health policies, and no legislation prohibits political contributions from the tobacco industry. More efforts in the systematic implementation of Article 5.3 of the WHO FCTC are needed in the country in the next years.

Our analysis revealed an overall declining trend in smoking prevalence among both sexes and across all age groups. However, the persistently high prevalence, recent upward shifts in certain subgroups, and the growing use of alternative nicotine products raise public health concerns and underscore the need for targeted interventions. Public health authorities should implement immediate measures, including stronger prevention policies, taxation, regulation of alternative products, and advertising restrictions.

### Limitations

This study lacks data for the year 2004, interrupting the continuity of the temporal analysis. However, this gap exists because the national survey was not conducted that year. Despite this missing data point, the Joinpoint analysis still allows the assessment of overall trends.

Second, the study analyse only aggregated data at the population level since no individual-level data were available. This study could be subject to the ecological fallacy, as the population-level data cannot capture individual behaviors or outcomes, potentially leading to overgeneralized inferences. Moreover, socio-economic determinants of smoking were not examined.

Third, although the data show clear changes in smoking patterns following the implementation of the Law, the analysis cannot statistically confirm a direct causal relationship.

Finally, the ecological time-series design does not allow disentangling age, period, and cohort effects, and some trends may partly reflect cohort-related differences in smoking behaviors.

The study may underestimate the role of emerging tobacco consumption patterns, particularly regarding the rapid proliferation of new products such as disposable vapes and the limited availability of data on HTPs use, currently covering only a short observation period. However, the Italian Ministry of Health has only recently begun collecting data on these new devices, and therefore, long-term trends are not yet available.

## Conclusions

Despite substantial progress in reducing smoking prevalence over the past three decades, our findings highlight that tobacco use remains a major public health concern in Italy. The recent resurgence in smoking, particularly among young adults and older adults, together with the increasing diffusion of alternative nicotine products, calls for renewed and targeted interventions. The misconceptions about the safety of novel tobacco substitutes needs immediate evidence-based communication campaigns. A more consistent implementation of the WHO FCTC, maintaining vigilance and adapting tobacco control policies to evolving consumption patterns remains essential to ensure continued progress toward a smoke-free society.

## Data Availability

The data used in this study are publicly available on the ISTAT website available at https://www.istat.it/sistema-informativo-6/health-for-all-italia/.

## Notes

**Michele Bonacquisti**, MD, Resident in Hygiene and Preventive Medicine

**Luigi Russo**, MD, Resident in Hygiene and Preventive Medicine

**Maria Rosaria Gualano**, MD, PhD, Associate Professor of Hygiene and Preventive Medicine

**Walter Ricciardi**, MD, Professor of Hygiene and Preventive Medicine

**Leonardo Villani**, MD, PhD, Associate Professor of Hygiene and Preventive Medicine
